# Reduced Cytokine Release in Ex Vivo Response to Cilengitide and Cetuximab Is a Marker for Improved Survival of Head and Neck Cancer Patients

**DOI:** 10.3390/cancers9090117

**Published:** 2017-09-05

**Authors:** Susan Cedra, Susanne Wiegand, Marlen Kolb, Andreas Dietz, Gunnar Wichmann

**Affiliations:** Department of Otolaryngology, Head and Neck Surgery, University of Leipzig, 04103 Leipzig, Germany; scedra@gmx.net (S.C.); Susanne.Wiegand@medizin.uni-leipzig.de (S.W.); Marlen.Kolb@medizin.uni-leipzig.de (M.K.); andreas.dietz@medizin.uni-leipzig.de (A.D.)

**Keywords:** head and neck cancer, head and neck squamous cell carcinoma (HNSCC), predictive assay, chemoresponse ex vivo, cilengitide, integrin, α_V_β3, targeted therapy, biomarker, interleukin 6, monocyte chemotactic protein-1

## Abstract

Targeting of α_V_β3 and α_V_β5 integrins by cilengitide may reduce growth of solid tumors including head and neck squamous cell carcinoma (HNSCC). Preclinical investigations suggest increased activity of cilengitide in combination with other treatment modalities. The only published trial in HNSCC (ADVANTAGE) investigated cisplatin, 5-fluorouracil, and cetuximab (PFE) without or with once (PFE+CIL1W) or twice weekly cilengitide (PFE+CIL2W) in recurrent/metastatic HNSCC. ADVANTAGE showed good tolerability of the cilengitide arms and even lower adverse events (AEs) compared to PFE but not the benefit in overall survival expected based on preclinical data. As we found in the FLAVINO assay, a short-time ex vivo assay for prediction of chemosensitivity, only a subgroup of HNSCC had an increased suppressive effect of cilengitide containing combination therapies on colony formation of epithelial cells (CF_ec_) and release of pro-angiogenetic and pro-inflammatory cytokines, whereas other HNSCC failed to respond. Response to α_V_β3 and α_V_β5 integrin targeting by cilengitide classifies HNSCC regarding outcome. We present FLAVINO data arguing for further development of cilengitide plus cetuximab in treatment of a subgroup of HNSCC potentially identified by the FLAVINO assay using a set of biomarkers for response evaluation.

## 1. Introduction

As reviewed by Ahmedah and colleagues earlier this year, α_V_ integrins play a crucial role in the development, progression, and metastatic spread of head and neck squamous cell carcinoma (HNSCC), thus supporting the therapeutic potential of integrin targeting [1].

In our translational study, we focused on the efficacy of cilengitide applied either alone or as part of multi-component chemotherapy in HNSCC ex vivo. Here we present our findings including the involvement of pro-inflammatory and pro-angiogenic cytokines as potential prognostic markers.

Cilengitide, an N-methylated cyclic pentapeptide (cyclo-Arg-Gly-Asp-D-Phe-(N-methyl)-Val; EMD 121974; Merck KGaA, Darmstadt, Germany) containing the characteristic RGD-recognition sequence, specifically inhibits integrins α_V_β3 and α_V_β5 [2,3,4]. In the ex vivo short-time chemoresponse assay FLAVINO, we used alterations in colony formation and cytokine release of interleukin 6 (IL-6) and monocyte chemotactic protein-1 (MCP-1) as read-out for response of HNSCC to cilengitide plus cetuximab.

IL-6 is a pleiotropic pro-inflammatory cytokine involved in numerous biological processes such as cell differentiation, wound healing, and apoptosis [5]. Dysregulated or excessive expressed IL-6 is associated with a variety of chronic inflammatory diseases and the development and maintenance of malignant tumors including HNSCC [6]. Elevated IL-6 levels in patients with HNSCC correlate with higher tumor stage, lymph node metastasis, increased proliferative tumor-activity, decreased immunologic response, and distinctive cachexia [7]. Furthermore, IL-6 supports vascular endothelial growth factor A production, resulting in enhanced tumor-angiogenesis [8]. There is evidence that IL-6 concentration decreases during successful anti-tumoral therapy, suggesting predictive qualities of the course of IL-6 concentration alteration [7].

As shown in previous studies by our laboratory, cilengitide modulates effects of MCP-1 production in HNSCC ex vivo [9,10], thus indicating MCP-1 as a valid molecule for testing. MCP-1 is a pleiotropic chemotactic cytokine that is essential for the recruitment of monocytes, macrophages, and natural killer cells during tissue injury and inflammation [11]. It is known that high concentrations of this CC-chemokine have impact on tumor environment and that it is intertwined with tumor invasiveness, progress of disease, metastatic spread, and tumor-angiogenesis [12,13]. MCP-1 demonstrates both tumor-supporting and anti-tumor effects due to its diverse influences on different cell types, but it is still not clear how it decides to act either way—probably concentration dependency plays a leading role in this question [10]. Many studies have linked elevated MCP-1 serum levels in patients with the presence, development, or recurrence of solid tumors, including advanced HNSCC, with significant lower overall survival (OS) and tumor-specific survival (TSS), indicating MCP-1 as a prognostic biomarker for solid tumors including HNSCC and clinical outcome [14].

## 2. Results

In analyses including specimens of 39 HNSCC patients (Table 1) we found a tremendous heterogeneity in the release of the cytokines IL-6 and MCP-1 after short-time culturing (72 h; see Material and Methods Section) of HNSCC.

**Figure 1:**
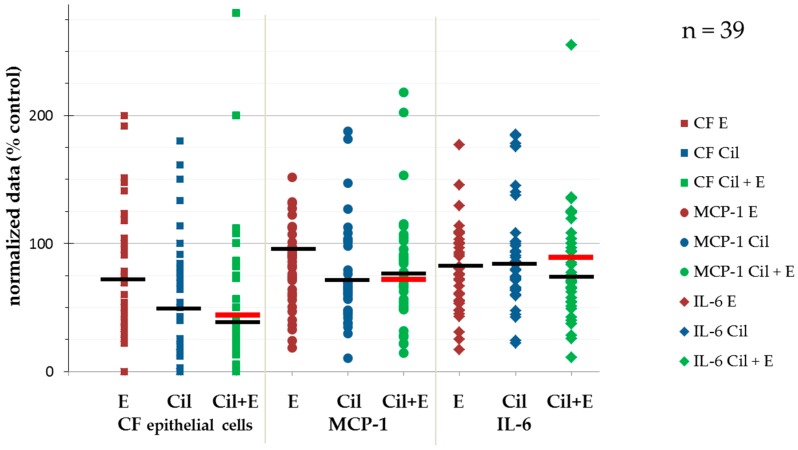
Heterogeneity in response of 39 HNSCC in the FLAVINO assay according to the readouts of colony formation (CF), release of MCP-1, and interleukin 6. Black lines represent median values, red lines represent cutoff values identified in receiver operator characteristic (ROC) analyses (Figures S1–S3). Abbreviations: CF, colony formation; IL-6, interleukin 6; MCP-1, monocyte chemotactic protein-1; Cil, cilengitide; E, Erbitux (cetuximab); Cil+E, cilengitide combined with Erbitux (cetuximab).

This is consistent with earlier findings in investigations applying cilengitide either alone or in combination with other treatments to solid tumors in vitro and in vivo [15,16,17,18,19,20,21,22,23]. We also detected varying efficacy regarding reduction of colony formation. The heterogeneity in response of HNSCC to cilengitide and in particular to cilengitide in binary combination with cetuximab (Cil+E) is substantial (Figure 1).

**Figure 2:**
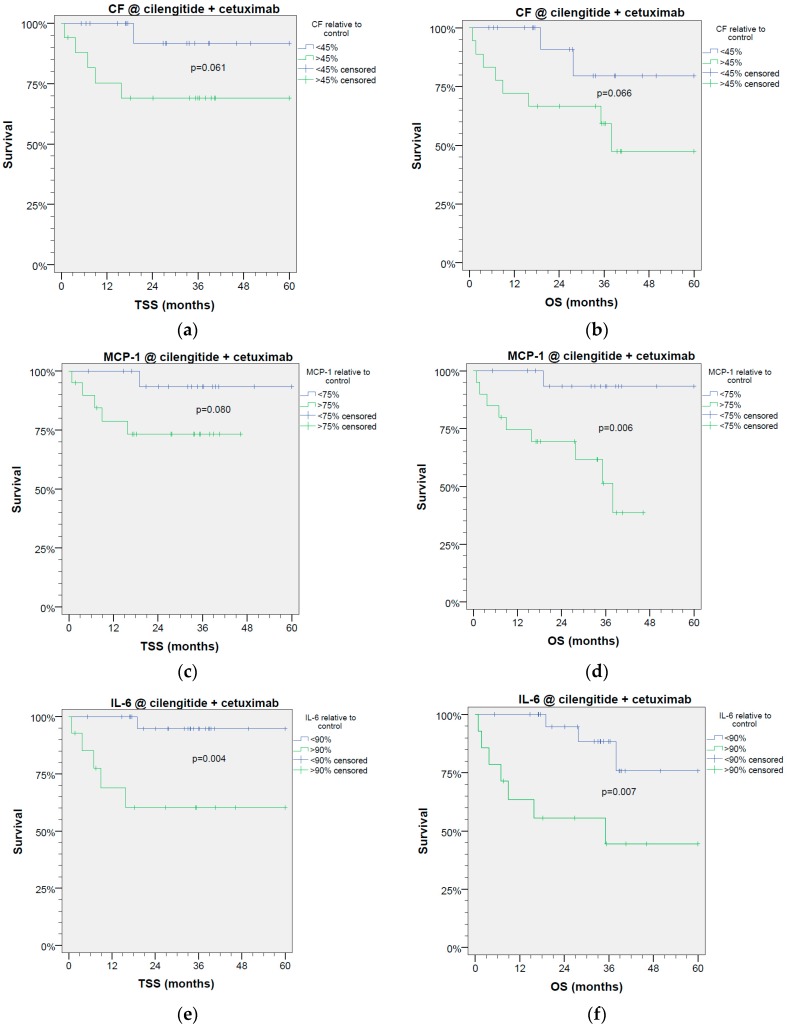
Colony formation of epithelial cells (CFec) and release of MCP-1 and IL-6 are prognostic factors for tumor-specific (**a**,**c**,**e**) and overall survival (**b**,**d**,**f**) of HNSCC patients. (**a**,**b**) Colony formation; (**c**,**d**) release of MCP-1, (**e**,**f**) release of IL-6 as optimized classifiers for survival derived from significant receiver-operating characteristic curves obtained from *n* = 39 HNSCC patients.

We analyzed the outcome of patients under study regarding OS using receiver operator characteristic (ROC) curves (Supplementary Figures S1–S3) and found significant areas under the curve (AUC) regarding predicted survival for MCP-1 ≤ 75%, and statistical trends for CF ≤ 45%, and IL-6 ≤ 90% of controls. The grouping of HNSCC patients according to these cutoff values revealed significantly different TSS and OS of patients (Figure 2). Suppression of MCP-1 concentration below 75% of controls and IL-6 concentration below 90% of controls under treatment of cetuximab plus cilengitide were associated with significantly improved OS (MCP-1 *p* < 0.006; IL-6 *p* < 0.007) and TSS (MCP-1 *p* < 0.08; IL-6 *p* < 0.004). This trend for improved survival was also seen in patients whose HNSCC responded to Cil+E with suppression of CF_ec_ below 45% of controls (OS *p* < 0.066; TSS *p* < 0.061). Since readouts of cytokine release in triplicate measurements achieved more consistent and objective results than manual microscopic cell counting for CF_ec_ [9], measurement of IL-6 and MCP-1 might be a suitable new strategy for the prediction of patients’ likely outcomes. Independent from the chosen readout, the statistical analyses using Fisher’s exact test revealed no significant difference in distribution of values above and below the cutoff regarding response of HNSCC samples taken from early stages (UICC I and II) vs. locoregional advanced HNSCC stages (UICC III and IV) or T categories T1 and T2 vs. T3 and T4 to either cilengitide, cetuximab, or Cil+E and the detected prognostic value (all *p* > 0.387). Only slight and insignificant differences in the patients’ OS associated with UICC stage and T category were found in Kaplan Meier curves comparing binary classified patients according to the cutoff for the readouts CFec (*p* = 0.951 and *p* = 0.465), or the release of IL-6 (*p* = 0.955 and *p* = 0.501) or MCP-1 (*p* = 0.883 and *p* = 0.771).

## 3. Discussion

Our set of data demonstrates a huge heterogeneity of head and neck cancer cells in response to Cil+E. A strongly reduced release of IL-6 and MCP-1 by Cil+E treated HNSCC is demonstrated as being a potential classifier for the patients’ OS and TSS. Our results also imply the existence of a subgroup of patients potentially benefitting from combined Cil+E treatment.

A more detailed look at results obtained in ADVANTAGE [24,25] reveals a 10% higher objective response rate (ORR) in PFE + CIL1W vs. PFE of 42% (95% confidence interval, CI, 30–55%) vs. 32% (95% CI 21–45%) corresponding to an odds ratio (OR) of 1.516 (95% CI 0.732–3.141) in the independent read (sensitivity analysis). This higher ORR in PFE+CIL1W vs. PFE was accompanied by a prolonged median time to treatment failure (5.6 vs. 4.2 months) but led to a slightly increased hazard ratio (HR) for progression-free survival (PFS; HR 1.15, 95% CI 0.74–1.79; *p* = 0.528), whereas the OS of patients treated with PFE+CIL1W was not inferior (HR 0.94, 95% CI 0.61–1.47; *p* = 0.800). 

As the primary objective of the trial (i.e., reasonably improved OS) was not met, the investigators concluded that further development of PFE+CIL1W or PFE+CIL2W in R/M HNSCC cannot be recommended. Does this mean that also further development of the binary combination of cilengitide plus cetuximab for treatment of R/M HNSCC should not be recommended?

Our findings in the FLAVINO study [9] and the here shown suitability of these data to classify HNSCC regarding their prognosis together with the data published for ADVANTAGE strongly suggest considerable therapeutic potential of cilengitide in combination with cetuximab in preselected HNSCC patients potentially identified using the outcome measured in the FLAVINO assay. As cilengitide even in combination with multiple other chemotherapeutics was well tolerated [21,24], there might be a place for cilengitide in binary combination with cetuximab (Cil+E) instead of cetuximab monotherapy in R/M HNSCC without adequate general health to receive PFE or after failure of platinum-based regimens. Future research may reveal if cilengitide deserves another chance in R/M HNSCC.

## 4. Materials and Methods

### 4.1. Patient Characteristics

HNSCC samples of 43 patients were included in this study. With patients’ informed consent, biopsies of tumor tissue were obtained during surgery or panendoscopy. All patients received therapy according to the consented decision made by the Head and Neck Cancer Tumor Board of the University Hospital Leipzig according to German therapy guidelines; none of the patients were treated with cilengitide and/or cetuximab. Thirty-nine histopathologically confirmed HNSCC of these 43 patients, 34 male and 5 female patients (mean age of 60.3 years; Table 1), could be analyzed regarding cytokine production and colony formation after treating the specimens with either Cil or E alone or combined (Cil+E).

### 4.2. FLAVINO-Assay

The same procedures as the protocol of the FLAVINO assay were used, as previously described [9]. Freshly obtained tumor specimens were put into phenol red- and riboflavin-free medium supplemented with 10% fetal calf serum and antibiotics (TM). After mechanical disintegration and digestion by 230 mU/mL collagenase IV (Sigma–Aldrich, Deisenhofen, Germany), 10,000 viable HNSCC cells were added to triplicate wells coated with human laminin (Roche, Germany) containing either 66.7 µg/mL E, 10 µM Cil, Cil+E, or (for reference) TM alone, adjusting the total volume to 300 µL. Supernatants harvested after 3 days were analyzed by ELISA and adherent cells ethanol-fixated and underwent pan-cytokeratin staining by FITC-labeled antibodies and counting of green fluorescent colonies of epithelial cells. Thirty-nine HNSCC had adherent growth (mean CF_ec_ ≥ 4/well in triplicate controls).

### 4.3. ELISA

The cytokines IL-6 and MCP-1 released into supernatants were measured using indirect sandwich ELISAs (OptEIA Kits; BD Biosciences, Heidelberg, Germany) following the manufacturer’s instructions and using tetra-methyl benzidine as substrate. The optical density of each well was determined measuring the optical density at λ_1_ = 450 nm and λ_2_ = 620 nm on the Synergy2^TM^ multi-mode microplate reader (BioTek Instruments, Inc., Winooski, VT, USA) according to 4-parameter calibration curves calculated using Gen5 software (BioTek). The lower limit of detection (LLD) was ≤4 pg/mL, the lower limit of quantification (LLQ) was ≤7 pg/mL for both cytokines, two orders of magnitude below median cytokine concentrations in untreated controls [9].

### 4.4. Statistical Analysis

All data shown are based on triplicate measurements. Differences were compared by Student’s *t*-test for paired samples. The maximum Youden scores (sensitivity plus specificity) were used for finding in ROC curves with significant areas under the curve the optimum cutoff for binary classification of HNSCC patients for survival data analyses according to the Kaplan-Meier method by applying the log-rank test. Statistics were done using SPSS Statistics for Windows, version 20.0.0 (SPSS Inc., Chicago, IL, USA). *P* ≤ 0.05 was regarded as significant.

### 4.5. Ethical Approval

The study was approved by the Ethics Committee of the Medical Faculty of the University Leipzig (study codes 180-2007, 201-10-12072010, and 341-15-05102015) and performed in accordance with the ethical standards as laid down in the 1964 declaration of Helsinki and its later amendments.

## 5. Conclusions

In a subgroup of HNSCC, the ex vivo targeting α_V_β3 and α_V_β5 integrins by cilengitide and EGFR by cetuximab leads to reduced CF_ec_ and release of IL-6 and MCP-1, thus proving that cilengitide in binary combination with cetuximab does have potential in the treatment of this subgroup. As response in the FLAVINO assay was associated with response in vivo [9,26], the FLAVINO assay may allow for the detection of HNSCC patients that could benefit from the combined targeted therapy. In this respect, IL-6 and MCP-1 gain significance as prognostic biomarkers for improved overall survival when cetuximab plus cilengitide achieve a decrease of IL-6 production <90% and of MCP-1 production <75% relative to control.

Future research may focus on the development of cilengitide plus cetuximab in preselected R/M HNSCC.

## Figures and Tables

**Table 1:** Characteristics of 39 histopathologically confirmed head and neck squamous cell carcinoma (HNSCC) of 34 male and 5 female patients (mean age at 60.3 years).

	n	(%)		n	(%)
**Localization**			**Tumor Stage**		
hypopharynx/larynx	12	(30.8%)	UICC I	2	(5.1%)
oropharynx	24	(61.5%)	UICC II	6	(15.4%)
nasopharynx	1	(2.6%)	UICC III	7	(17.9%)
oral cavity	2	(5.1%)	UICC IV	24	(61.5%)
**T Category**			**Lifetime Tobacco Exposure (Pack Years, py)**		
T1	5	(12.8%)	0 py	8	(20.5%)
T2	7	(17.9%)	1–20 py	3	(7.7%)
T3	14	(35.9%)	21–40 py	14	(35.9%)
T4a	13	(33.3%)	41–60 py	8	(20.5%)
T4b	0	(0.0%)	>60 py	4	(10.3%)
			no information	2	(5.1%)
**N Category**			**Alcohol Consumption (g/day)**		
N0	16	(41.0%)	0	4	(10.3%)
N1	3	(7.7%)	<30	14	(35.9%)
N2a	1	(2.6%)	31–60	8	(20.5%)
N2b	6	(15.4%)	≥60	11	(28.2%)
N2c	12	(30.8%)	no information	2	(5.1%)
N3	1	(2.6%)			

## Supplementary Materials

The following are available online at http://www.mdpi.com/2072-6694/9/9/117/s1. Supplementary Figures S1–S3.
