# Supplementary material for: Reduced Cytokine Release in Ex Vivo Response to Cilengitide and Cetuximab Is a Marker for Improved Survival of Head and Neck Cancer Patients
Source: Cancers (Basel). 2017 Sep 5;9(9):117. doi: 10.3390/cancers9090117 (PMC5615332; doi:10.3390/cancers9090117)
Supplement: Supplementary file 1 [file cancers-09-00117-s001.docx]

Supplementary Materials: Reduced Cytokine Release in Ex Vivo Response to Cilengitide and Cetuximab Is a Marker for Improved Survival of Head and Neck Cancer Patients

Susan Cedra, Susanne Wiegand, Marlen Kolb, Andreas Dietz and Gunnar Wichmann


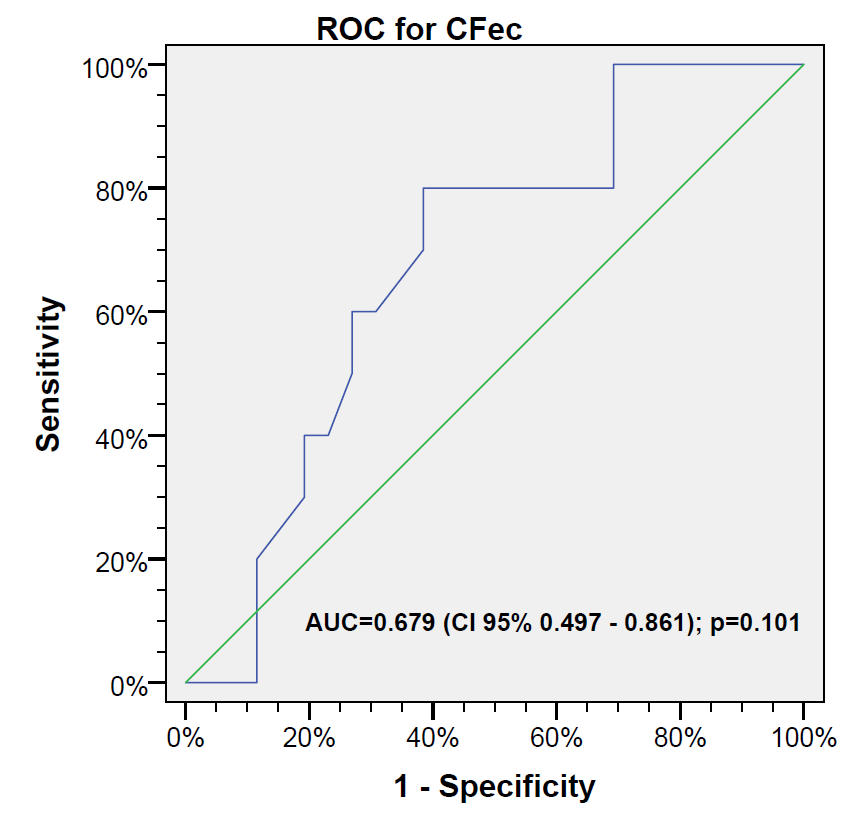


**Figure S1.** The ROC curve for colony formation of epithelial cells (CFec) from head and neck squamous cell carcinoma in the short-time ex-vivo assay FLAVINO shows an association of CFec and overall survival of HNSCC patients.


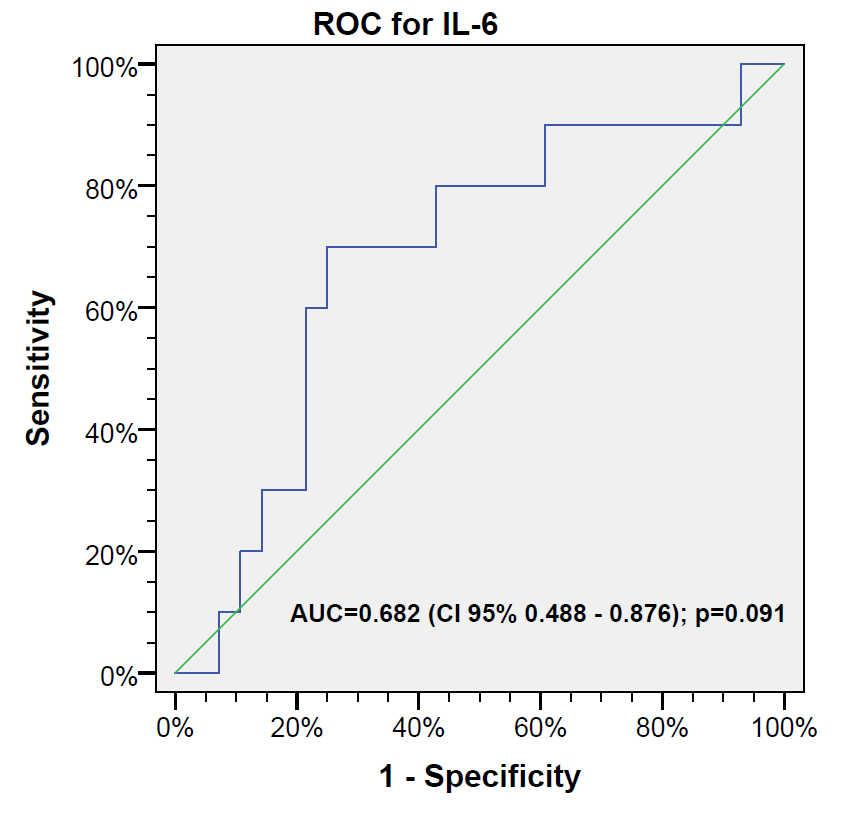


**Figure S2.** The ROC curve for release of interleukin 6 (IL-6) by head and neck squamous cell carinoma (HNSCC) cells treated with cetuximab plus cilengitide according to the protocol of the short-time ex-vivo assay FLAVINO shows an association of IL-6 release and overall survival of HNSCC patients.


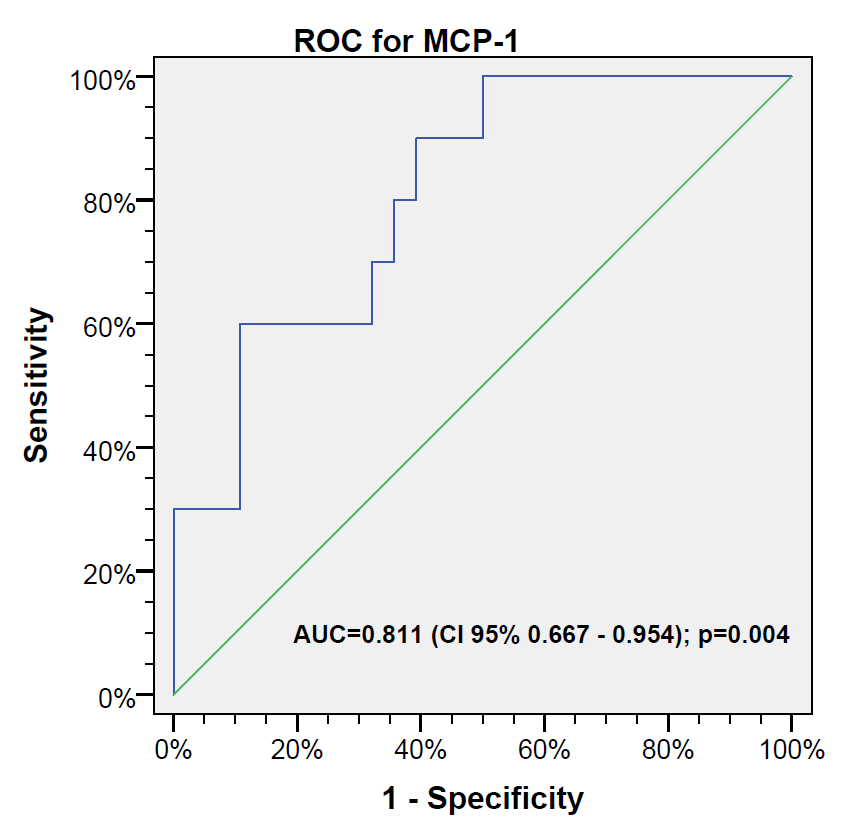


**Figure S3.** The ROC curve for release of monocyte chemoattractant protein-1 (MCP-1) by head and neck squamous cell carinoma (HNSCC) cells treated with cetuximab plus cilengitide according to the protocol of the short-time ex-vivo assay FLAVINO shows a significant association of MCP-1 release and overall survival of HNSCC patients.
